# Anti-tissue transglutaminase antibodies (TG2A) positivity and the risk of vitamin D deficiency among children - a cross-sectional study in the generation R cohort

**DOI:** 10.1186/s12887-023-04041-x

**Published:** 2023-06-07

**Authors:** Laura A. van der Velde, Sanne A. Beth, Trudy Voortman, Menno C. van Zelm, Henriette A. Moll, Jessica C. Kiefte-de Jong

**Affiliations:** 1 Health Campus The Hague/Department of Public Health and Primary Care, Leiden University Medical Center, The Hague, The Netherlands; 2 The Generation R Study Group, Erasmus MC, Rotterdam, The Netherlands; 3 Department of Pediatrics, Erasmus MC, Rotterdam, The Netherlands; 4 Department of Epidemiology, Erasmus MC, Rotterdam, The Netherlands; 5 Department of Immunology and Pathology, Central Clinical School, Monash University and Alfred Health, Melbourne, Australia

**Keywords:** Celiac disease, Celiac disease autoimmunity, Vitamin D, Vitamin D deficiency, Child, Children, Pediatric population

## Abstract

**Background:**

Suboptimal vitamin D status is common in people with celiac disease (CeD), a disease that can be characterized by the presence of serum anti-tissue transglutaminase antibodies (TG2A) (i.e., TG2A positivity). To date, it remains unclear whether childhood TG2A positivity is associated with vitamin D status and how this potential association can be explained by other factors than malabsorption only, since vitamin D is mainly derived from exposure to sunlight. The aim of our study was therefore to assess whether childhood TG2A positivity is associated with vitamin D concentrations, and if so, to what extent this association can be explained by sociodemographic and lifestyle factors.

**Methods:**

This cross-sectional study was embedded in the Generation R Study, a population-based prospective cohort. We measured serum anti-tissue transglutaminase antibodies (TG2A) concentrations and serum 25-hydroxyvitamin D (25(OH)D) concentrations of 3994 children (median age of 5.9 years). Children with serum TG2A concentrations ≥ 7 U/mL were considered TG2A positive. To examine associations between TG2A positivity and 25(OH)D concentrations, we performed multivariable linear regression, adjusted for sociodemographic and lifestyle factors.

**Results:**

Vitamin D deficiency (serum 25(OH)D < 50 nmol/L) was found in 17 out of 54 TG2A positive children (31.5%), as compared to 1182 out of 3940 TG2A negative children (30.0%). Furthermore, TG2A positivity was not associated with 25(OH)D concentrations (β -2.20; 95% CI -9.72;5.33 for TG2A positive vs. TG2A negative children), and this did not change after adjustment for confounders (β -1.73, 95% CI -8.31;4.85).

**Conclusions:**

Our findings suggest there is no association between TG2A positivity and suboptimal vitamin D status in the general pediatric population. However, the overall prevalence of vitamin D deficiency in both populations was high, suggesting that screening for vitamin D deficiency among children, regardless of TG2A positivity, would be beneficial to ensure early dietary intervention if needed.

**Supplementary Information:**

The online version contains supplementary material available at 10.1186/s12887-023-04041-x.

## Background

Celiac disease (CeD) is a common chronic immune-mediated enteropathy triggered by the ingestion of gluten in genetically-predisposed individuals [1]. The multifactorial etiology of the disease possibly involves a complex interplay between genetics, gluten intake, intestinal microbiota, and both innate and adaptive immune responses [2]. Approximately 1–3% of the general population across various geographic areas are diagnosed with CeD [3]. It is assumed that the actual prevalence is even higher, because the disease often elapses clinically atypical and therefore often remains undiagnosed [4]. CeD incidence has risen in the past decades [5]. This rise cannot be explained by genetic factors only, and therefore other factors such as environmental and lifestyle factors are expected to play an important role [4].

In addition to the wide range of symptoms of CeD, such as abdominal bloating and pain, low serum concentrations of vitamin D are often found at the moment of CeD diagnosis [6]. Vitamin D is known for its key role in bone mineral health and calcium homeostasis, as well as for its immune regulatory effect by modulating production of inflammatory cytokines and the proliferation of proinflammatory cells [6]. On the other hand, chronic inflammation of the small bowel in CeD leads to villous atrophy and, consequently, malabsorption of vitamin D may occur [7]. However, vitamin D is mainly produced in the skin upon sunlight exposure and to a lesser extent obtained from dietary intake [8]. Thus, other factors than malabsorption leading to low vitamin D concentrations should be considered, such as an increased catabolism of vitamin D due to the chronic inflammation, or insufficient sunlight exposure due to chronic illness [9]. However, as current recommendations indicate to limit sun exposure because of its association with skin cancer, dietary intake remains an important source of vitamin D [10].

Despite the unresolved issue of vitamin D deficiency being a risk factor or consequence of pediatric CeD [7, 11], Snyder et al. (2016) recently recommended to screen for vitamin D status at the time a diagnosis of CeD is considered [12]. Notwithstanding this, a concomitant vitamin D deficiency could further affect health in a child with CeD. Current guidelines indicate that CeD diagnosis requires serological testing and histological confirmation from small-bowel biopsies, a very invasive assessment. Fortunately, CeD can also be characterized by the presence of serum anti-tissue transglutaminase antibodies (TG2A) (i.e., TG2A positivity). A recent systematic review and meta-analysis indicated that TG2A has a sensitivity and specificity of over 90% for detection of small intestinal enteropathy [13]. Therefore, in the current study we used TG2A positivity as a reflection of potential CeD.

The aim of our study was to assess whether childhood TG2A positivity is associated with vitamin D concentrations, and if so, to what extent this association can be explained by sociodemographic and lifestyle factors.

## Methods

### Study design and population

This cross-sectional study was part of the Generation R Study, a multiethnic, population-based prospective cohort study performed in Rotterdam, the Netherlands. Mothers were invited to enroll with an expected delivery between 2002 and 2006, and their children have been followed from fetal life onwards (n = 9749). Detailed and extensive data were collected from the participants, including biological samples such as blood samples. All data collection and measurements were carried out in accordance with applicable guidelines and regulations and approved by the Medical Ethical Committee of Erasmus MC, University Medical Center Rotterdam. Further details on the design of the Generation R Study are described extensively elsewhere [14]. Written informed consent for participation was obtained from the parents of the participating children. The study was approved by the Medical Ethics Committee of the Erasmus Medical Center [15].

**Fig. 1:**
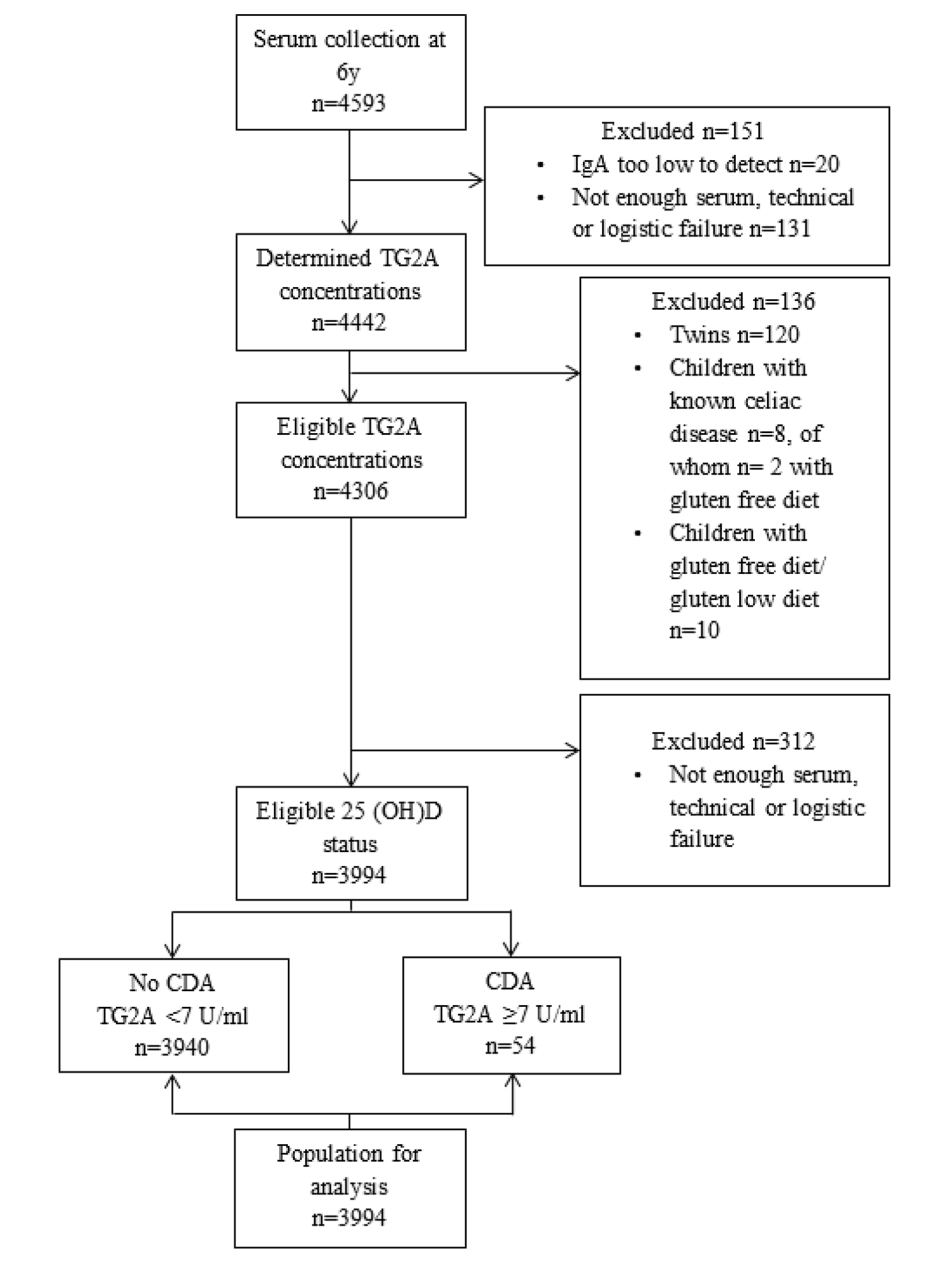
Flow chart of the study population selection for analysis: children’s TG2A concentrations and vitamin D status

At a median age of 5.9 years (IQR; 5.8-6.0), 6690 children visited the research center. Serum samples were collected from 4593 children. Subsequently, children were excluded from our analysis if: (1) insufficient amount of serum was available; (2) their IgA concentrations were below the detection limit (possibly IgA deficient); (3) they were twins (to avoid clustering of similar environmental and genetic risk of celiac disease); (4) they had an established CeD diagnosis; and/or (5) were on a gluten-free diet (Fig. 1). This resulted in a total study population of 3994 children, of whom we measured the serum anti-tissue transglutaminase antibodies (TG2A) concentrations and serum 25-hydroxyvitamin D (25(OH)D) concentrations.

### Anti-tissue transglutaminase antibodies (TG2A) positivity

Serum TG2A concentrations were determined using a fluorescence enzyme immunoassay (ELiA Celikey IgA, PhadiaImmunocap 250; EliA IgA, Phadia AB, Uppsala Sweden) at the Department of Immunology of the Erasmus MC [16]. Intra-essay and inter-assay coefficients of variability were < 10% and < 15%, respectively. Children with serum TG2A concentrations of < 7 U/ml were considered TG2A negative and children with TG2A concentrations of ≥ 7 U/ml were considered TG2A positive, as per the manufacturer’s instructions. TG2A positive children were offered further clinical follow-up at the Department of Pediatric Gastroenterology, Erasmus MC, Rotterdam.

To identify the genetic predisposition to celiac disease, an approximation of HLA-DQ2 or HLA-DQ8 carriership was determined using a tag single nucleotide polymorphism approach as described in detail earlier [16, 17]. Children were considered HLA-DQ2/DQ8 positive when at least 1 of the haplotypes was present.

### Vitamin D status assessment

Measurements of 25(OH)D concentrations were performed at the Endocrine Laboratory of the VU University Medical Center, Amsterdam. Isotope dilution on-line solid phase extraction liquid chromatography-tandem mass spectrometry (ID-XLC-MS/MS) was used, as described in detail earlier [18]. The limit of quantitation was 4.0 nmol/L, intra-assay and inter-assay coefficients of variation were < 6% and < 8%, respectively. On the basis of recommendations and previous studies in the pediatric population we defined 3 cut-offs for vitamin D status: deficient (< 50 nmol/L), sufficient (50 to < 75 nmol/L) and optimal (≥ 75 nmol/L) [19–25].

### Covariates

We identified potential covariates in the association between CeD and vitamin D status based on both existing literature [7, 9, 26–29] and previously reported predictors in our own study population [16, 18, 30]. Information on demographics, ethnicity and lifestyle characteristics were assessed using questionnaires, detailed physical and serological examinations, and medical records. Child’s age, sex and birth weight were obtained from obstetric records. From a subgroup, cord blood samples were collected at birth and a genome-wide association analysis (GWA) was done using Illumina Infinium II HumanHap610 Quad Arrays, following manufacturer’s protocols and imputed to the combined HapMap Phase II CEU, CHB/JPT, YRI panel [31]. To assess the risk for vitamin D deficiency, four SNPs involving vitamin D synthesis and metabolism were obtained from the GWA dataset: rs10741657 (near CYP2R1), rs12785878 (near DHCR7), rs2282679 (near GC) and rs6013897 (near CYP24A1) [28]. We determined child’s ethnicity by country of birth of the parents [32–34], and classified ethnicity as Western or non-Western, based on the geographical distribution of CeD and according to the criteria we used in a previous study [30]. Data on breastfeeding exclusiveness and timing of gluten introduction were obtained from delivery reports and postnatal questionnaires at 2, 6 and 12 months. Dietary intake was assessed with a semi-quantitative food frequency questionnaire (FFQ) at around the age of 1 year. To assess the quality of the children’s diets, we applied a previously developed diet quality score reflecting adherence to dietary guidelines for young children [35]. Diet quality scores were categorized as high and low diet quality scores, based on the population mean score. Data on the use of vitamin D supplementation at the age of 1 year were obtained from the same FFQ. Information on gastroenteritis was assessed by questionnaires and based on the report of fever combined with diarrhoea during the year before the 6-year visit to the research center. At the age of 6 years, anthropometric data were collected during the children’s visit to the research center. The season of blood draw was based on the date of the visit and categorized into winter/spring and summer/autumn. Information on the amount of playing outside during daytime was obtained from a questionnaire filled out by the parents.

Maternal educational level was obtained at enrollment and categorized into low (≤ secondary education) and high (≥ higher education). Smoking during pregnancy (yes or no) was assessed by prenatal questionnaires. During the 2nd trimester of pregnancy, serum samples were collected and 25(OH)D concentrations were obtained from 3039 mothers.

### Statistical analysis

Chi-square tests (for categorical variables), Mann-Whitney U tests (for continuous non-normally distributed variables), and Student’s T tests (for continuous normal distributed variables) were performed to compare the characteristics of TG2A negative (i.e., serum TG2A concentrations of < 7 U/ml) and TG2A positive (i.e., serum TG2A concentrations of ≥ 7 U/ml) children. Fisher’s exact test was obtained in case the expected count was less than five. Associations between TG2A positivity and vitamin D concentrations were examined using linear regression analysis, with serum 25(OH)D concentrations as continuous dependent variable and TG2A positivity (TG2A negative and TG2A positive) as binary independent variable. Covariates with p *<* 0.10 or with a change in effect estimate ≥ 10% in univariable regression analysis, were considered as potential confounding variables [36]. We performed three final regression models: a crude model, a multivariable model adjusted for season of blood draw and ethnicity (model 1) and a multivariable model adjusted for season of blood draw, ethnicity, serum 25(OH)D concentrations of the mother during pregnancy, child’s birth weight, gender and breastfeeding exclusiveness (model 2). In addition, analyses were performed to test whether the association between TG2A positivity and 25(OH)D concentrations was different for HLA DQ2/DQ8 positive versus HLA DQ2/DQ8 negative children.

Sensitivity analyses were performed to test whether the association between TG2A positivity and 25(OH)D concentrations was different for children with high versus low diet scores (based on the mean diet score). Furthermore, we investigated possible effect modification by the four different vitamin D deficiency risk alleles.

To reduce attrition bias, covariates were imputed (*n* = 5 imputations), using the Fully Conditional Specification method. Pooled results of the imputed data were presented. Regression coefficients were pooled by taking the average of the coefficients of the five datasets. The pooled results of the regression analysis were presented as beta’s and 95% confidence intervals (CIs). All statistical analyses were performed using the software package IBM SPSS Statistics, version 22.0, for Windows (IBM Corp, Armonk, NY).

## Results

### Subject characteristics

Out of 3994 children tested, 54 (1.4%) wereTG2A positive (Fig. 1). Of them, 51 children agreed on further clinical follow-up at the Department of Pediatric Gastroenterology. Thirty-one children (60.8%) had CeD, 10 (19.6%) did not have CeD, and 10 (19.6%) were considered potential CeD cases because of inconclusive serologies [37]. TG2A positive children more often were girls (64.8% vs. 48.2%), had on average a lower BMI (15.6 kg/m^2^ vs. 16.2 kg/m^2^) (Table 1**)**, and were more often HLA DQ2 and/or HLA DQ8 positive (89.7% vs. 41.1%) (Supplementary Table 1). Of the n = 1105 HLA DQ2 and/or HLA DQ8 positive children, 1070 children (96.8%) were TG2A negative, and 35 children (3.2%) were TG2A positive (data not shown).

**Table 1:** Characteristics of the children and their mothers, for the total population and split by TG2A positivity (imputed data)

	Total population (n = 3994)	TG2A positivity at 6 years of age		
		**TG2A negative** (TG2A < 7 U/ml) (n = 3940)	**TG2A positive** (TG2A ≥ 7 U/ml) (n = 54)	**p** ***-*** **value** ^**a**^
**Child characteristics** ^**b**^				
Birth weight (grams; mean, SD)	3434 (551.6)	3433.2 (551.0)	3493.7 (604.6)	0.42
Female gender (n,%)	1934 (48.4)	1899 (48.2)	35 (64.8)	**0.01**
Ethnicity (n,%)				
Non-Western	1364 (34.2)	1352 (34.3)	12 (22.2)	0.08
Western	2630 (65.8)	2588 (65.7)	42 (77.8)	
Breastfeeding (n,%)				
Exclusively breastfed in the first 4 months	1302 (32.6)	1282 (32.1)	20 (37.0)	0.21
Partially breastfed in the first 4 months	2354 (58.9)	2320 (58.1)	33 (61.1)	
Never breastfed	338 (8.5)	337 (8.4)	1 (1.9)	
Gluten introduction > 6 months (n,%)	2143 (53.7)	2117 (53.1)	26 (48.1)	0.43
Gastroenteritis in the past year (n, %)	1712 (42.9)	1686 (48.3)	26 (48.1)	0.48
BMI child at 6 years of age (kg/m^2^; median, IQR)	16.2 (15.1–16.9)	16.2 (15.1–16.9)	15.6 (14.7–16.3)	**0.01**
Playing outside at 6 years of age (hours/ day; median, IQR)	1.6 (0.8–2.1)	1.6 (0.8–2.1)	1.4 (0.7-2.0)	0.78
**Maternal characteristics** ^**b**^				
Maternal educational level (n,%)				
Low (≤ secondary education)	2127 (53.3)	2104 (52.7)	23 (42.6)	0.21
High (≥ higher education)	1867 (46.7)	1836 (47.3)	31 (57.4)	
Maternal smoking during pregnancy (n,%)	641 (16.0)	633 (15.9)	8 (14.8)	0.70
Maternal 25(OH)D serum concentrations second trimester pregnancy (nmol/L; median, IQR)	53.1 (27.3–76.0)	53.2 (27.9–75.6)	53.3 (28.0-79.1)	0.72

Values are frequencies (percentages), means (standard deviations (SD)), or medians (interquartile range (IQR)) from the imputed dataset

^a^ p-values are calculated with the Chi-square test for categorical variables, Mann-Whitney U test for continuous non- normally distributed variables and Student’s T test for continuous normal distributed variables and reflect differences between TG2A positive and negative groups. Fisher’s exact test was obtained in case the expected count was less than five. A p-value < 0.05 was considered significant

^b^ Data are pooled results after multiple imputation

### TG2A positivity and vitamin D status

Child and maternal characteristics did not differ between TG2A negative and TG2A positive children, except for gender (TG2A positive children more often were female) and BMI at 6 years of age (TG2A positive children had a slightly lower BMI at 6 years of age) (Table 1).

At the age of 6 years, median 25(OH)D serum concentrations in the TG2A negative group was 63.9 (IQR: 45.1–81.0; rage: 4.1 to 211) nmol/L, as compared to 62.0 (IQR: 44.9–82.7; range: 15 to 131) nmol/L in the TG2A positive group (p = 0.8) (Supplementary Table 1).

Vitamin D status did not differ between TG2A negative and TG2A positive children. Of TG2A negative children, 30% had deficient vitamin D concentrations, 36.8% had sufficient vitamin D concentrations and 33.2% had optimal vitamin D concentrations. In the TG2A positive group, 31.5% of the children was vitamin D deficient, 27.8% had sufficient vitamin D concentrations and 40.7% had optimal vitamin D concentrations, respectively. (Supplementary Table 1).

In univariable regression analysis with 25(OH)D concentration as outcome, no association was found with TG2A positivity at 6 years of age (β= -2.20 nmol/L, 95% CI -9.72; 5.33). Results did not change after adjustment for season of blood draw and ethnicity (multivariable model 1: β= -2.20 nmol/L, 95% CI -8.73; 4.33), nor after additional adjustment for maternal 25(OH)D concentrations during pregnancy, birth weight, gender, and breastfeeding exclusiveness (multivariable model 2: β= -1.73 nmol/L, 95% CI -8.31; 4.85) (Table 2). Similar results were found for the associations between TG2A positivity and 25(OH)D concentrations in crude data (Supplementary Table 2). Also no associations were found between TG2A positivity and 25(OH)D concentrations separately adjusted for season of blood draw and ethnicity, maternal 25(OH)D concentrations during pregnancy, birth weight, gender, or breastfeeding exclusiveness in crude and imputed data (Supplementary Tables 3 and 4). Analyses stratified on HLA DQ2/DQ8 haplotype positivity showed similar results to those for the total study population (Table 2 and Supplementary Table 3).

**Table 2:** Associations between TG2A positivity (binary independent variable) and serum 25(OH)D concentrations (continuous dependent variable) at 6 years of age, for the total population and for HLA DQ2 and/or HLA DQ8 positive children (imputed data)

	**25 (OH) D concentrations (nmol/L)**		
**TG2A concentrations U/ml**	**Crude model** β (95% CI)	**Model 1** aβ (95% CI)	**Model 2** aβ (95% CI)
**TG2A negative** *n = 3940*	*Reference*	*Reference*	*Reference*
**TG2A positive** *n = 54*	-2.20 (-9.72;5.33)	-2.20 (-8.73;4.33)	-1.73 (-8.31;4.85)
	**25 (OH) D concentrations (nmol/L)**		
**TG2A concentrations U/ml among HLA DQ2 and/or HLA DQ8 positive children**	**Crude model** β (95% CI)	**Model 1** aβ (95% CI)	**Model 2** aβ (95% CI)
**TG2A negative** *n = 1070*	*Reference*	*Reference*	*Reference*
**TG2A positive** *n = 35*	-1.17 (-10.43;8.09)	-2.11 (-10.14;5.91)	-1.19 (-9.05; 6.66)

Values (β’s) are based on linear regression models and reflect between group differences in serum 25 (OH) D concentrations (nmol/L) at 6 years of age relative to the reference group.

*Denotes statistical significance (P < 0.05)

Model 1 was adjusted for season of blood draw (winter/spring and summer/autumn), and ethnicity (Western and non-Western). Model 2 was adjusted for season of blood draw, ethnicity, 25 (OH) D concentrations of the mother during pregnancy (nmol/L), child’s birth weight (grams), gender (male/ female) and breastfeeding exclusiveness (yes/no).

Abbreviations: β, beta; aβ, adjusted beta; CI, confidence interval.

In sensitivity analyses, no associations were found between TG2A positivity and 25(OH)D concentrations stratified on mean diet score (i.e., diet quality scores < 4.17 and ≥ 4.17), although a trend was observed towards lower 25(OH)D concentrations among those with TG2A positivity and lower diet quality scores (Supplementary Table 5a and 5b). Additional adjustments for body mass index (BMI; kg/m^2^) did not change our results (data not shown). No interactions were found for allele frequencies associated with vitamin D deficiency (Supplementary Table 6).

## Discussion

This study does not confirm the hypothesis that TG2A positivity is associated with suboptimal vitamin D status in the general pediatric population. Although we observed no association between vitamin D concentrations and TG2A positivity in children, we did find a high overall prevalence of vitamin D deficiency in both children with and without TG2A positivity.

Our results showed a high overall prevalence of vitamin D deficiency in children, regardless of TG2A positivity. This finding is in accordance with the current reports on vitamin D deficiency as a worldwide phenomenon [11, 18, 38, 39]. However, our results suggested no association between childhood TG2A positivity and vitamin D status. This is in line with the results of Villanueva et al., who found no difference in mean vitamin D concentrations between children with CeD and their controls [7]. This might be explained by our focus on the pediatric population, as previous research by Lerner et al. (2011) studying childhood and adulthood CeD in two countries with relatively high amounts of sunshine indicates a negative correlation between age and vitamin D status, unrelated to CeD and intestinal injury or inflammation [11]. They found that vitamin D concentrations were not different between the individuals with or without CeD, making it unlikely that the intestinal inflammation and malabsorption in CeD affected serum vitamin D concentrations as all participants were on a gluten containing diet [11].

It is important to note that sunlight is the main source of vitamin D (i.e., rather than dietary intake and dietary supplements), as vitamin D is made in the skin from exposure to sunlight [9]. Indeed, sun exposure is one of the main conditions affecting vitamin D status [11]. This might help explain the negative correlation between age and vitamin D status, as children are often more exposed to sunlight because they are unaware of the harmful effects of sun exposure [11].

Although several other studies showed that CeD is frequently associated with vitamin D deficiency [6, 40, 41], the potential role of vitamin D in the pathogenesis of CeD remains unclear. Furthermore, if an association is found, it remains of debate whether CeD is the consequence or the cause in relation to vitamin D deficiency. Recently, Wessels et al. reported that vitamin D status in relation to CeD might depend on more than a gluten free diet and vitamin D supplementation, since two-thirds of their patients remained vitamin D deficient after five-year follow up despite a prescription for vitamin D supplementation [42]. Interestingly, a study by Aronsson et al. (2021) showed a U-shaped relation between vitamin D concentrations and risk of CeD, whereas our results showed no indication for non-linearity [43]. Specifically, they found that besides low vitamin D concentrations also vitamin D concentrations above 75 nmol/L, most likely due to frequent vitamin D supplementation, were associated with an increased risk of developing CeD, which might be explained by upregulation of Th2 cell cytokines associated with immune reaction to external stimuli by high doses of vitamin D [43]. Our results do not support this hypothesis as we found no indication for non-linearity. To date, no clear consensus is reached regarding the optimal vitamin D concentration in childhood [43].

Children with CeD generally have suboptimal bone health, and clinical predictors for low bone mineral density in this population are currently lacking [44, 45]. Vitamin D is mainly known for its essential role in bone mineral health, by maintaining the homeostasis of calcium and phosphate [9, 46, 47]. It is thought that intestinal malabsorption due to chronic inflammation and villous atrophy is an important causative factor for vitamin D deficiency in CeD, and consequently might be one of the main causes of low bone mineral density (BMD) among children with CeD [6, 26, 40, 45, 46, 48]. Although calcitriol is the biologically active form of vitamin D, the total serum concentration of 25(OH)D is usually measured to determine vitamin D status and also considered a better measure, because of its higher concentrations and longer half-life [9, 40, 49]. However, concerning vitamin D status in CeD, it might be of importance to measure serum calcitriol concentrations as well, because low 25(OH)D does not necessarily reflect a deficiency of calcitriol and thus calcium imbalance [49, 50]. Furthermore, the question remains whether a strict association exists between intestinal vitamin D absorption and serum vitamin D concentrations. Vitamin D deficiency has been reported in CeD patients with normal intestinal vitamin D absorption, whereas patients with severely impaired intestinal vitamin D absorption showed normal serum vitamin D concentrations [9]. Schøsler et al. found decreased BMD in children with CeD, whereas not all children were vitamin D deficient [26]. An explanation for these findings might be that calcium malabsorption in CeD (and consequently impaired bone mineral health) may not be the result of vitamin D deficiency, but rather of a reduced level of calcium binding proteins due to intestinal villous atrophy [49]. Our results also do not support the hypothesis that decreased BMD in children with CeD is associated with vitamin D status, as our results showed no association between TG2A positivity and suboptimal vitamin D status.

### Methodological considerations

A major strength of our study is the large population-based prospective design. We measured both TG2A and 25(OH)D concentrations in a large group of children and we had information available on a wide range of child and maternal characteristics, allowing us to correct our analyses for multiple potential confounding factors. Another strength of our study is that children were unaware of the TG2A and 25(OH)D status at the time of visit to the research center, which minimises the risk of selection bias. Furthermore, we compared TG2A positive children with TG2A negative children. Several studies on vitamin D deficiency in relation to CeD are conducted without a comparison group [26, 41], which may lead to an overestimation of vitamin D deficiency because of intestinal malabsorption because vitamin D deficiency is already highly prevalent in the general population.

However, some limitations should be taken into account. Firstly, TG2A positivity ideally would have been complemented by additional measures (i.e., characteristic histological changes in the duodenal biopsy) to diagnose CeD. However, we were able to include HLA-DQ2/DQ8 positivity (reflecting genetic predisposition to celiac disease) and the diagnostic accuracy of serum TG2A concentration measurements is high. Therefore, for the aim of our study, TG2A positivity provided a suitable proxy for potential CeD [51]. Unfortunately, our study lacks information on serum parathyroid hormone (PTH) concentrations and thus we cannot say whether low vitamin D concentrations in TG2A positive children are likely to be associated with impaired bone mineral health. Furthermore, vitamin D serum concentration at 6 years of age was measured only once, whereas serum concentrations fluctuate over time. However, largest within-person fluctuations would be expected due to seasonality, which we adjusted for in our analyses. Further, vitamin D serum concentrations are influenced by dietary or supplemental vitamin D intake, of which we had no specific information available. We did however have information on overall dietary quality, which reflects adherence to dietary guidelines and therefore is a proxy for nutrient (and vitamin D) adequacy. Furthermore, a previous study by Voortman et al. (2015) has shown that diet quality in early childhood indeed was associated with vitamin D status at the age of 6 years [18].

## Conclusion

We found no association between TG2A positivity and suboptimal vitamin D status in the general pediatric population. We did, however, find a high overall prevalence of vitamin D deficiency in both populations, suggesting that screening for vitamin D deficiency among children, regardless of TG2A positivity, would be beneficial to ensure early dietary intervention if needed.

## Electronic supplementary material

Below is the link to the electronic supplementary material.

Supplementary Material 1

## Data Availability

The datasets used and/or analysed during the current study are available from the corresponding author on reasonable request.

## Abbreviations

CeD: celiac disease
CI: confidence interval
TG2A: anti-tissue transglutaminase antibodies
25(OH)D: 25-hydroxyvitamin D
β: beta
IQR: inter quartile range
IgA: immunoglobulin A
HLA: human leukocyte antigen
GWA: genome-wide association
FFQ: food frequency questionnaire
BMI: body mass index
PTH: parathyroid hormone
