# Supplementary material for: Anti-tissue transglutaminase antibodies (TG2A) positivity and the risk of vitamin D deficiency among children - a cross-sectional study in the generation R cohort
Source: BMC Pediatr. 2023 Jun 7;23:286. doi: 10.1186/s12887-023-04041-x (PMC10246407; doi:10.1186/s12887-023-04041-x)
Supplement: Supplementary file 1 — Supplementary Material 1 [file 12887_2023_4041_MOESM1_ESM.pdf]

Supplemental material for the manuscript entitled

'Anti-tissue transglutaminase antibodies (TG2A) positivity and the risk of vitamin D deficiency among children - A cross-sectional study in the Generation R cohort'

Supplementary Table 1. Characteristics of the children and their mothers, for the total population and split by TG2A positivity (observed data)

		TG2A positivity at 6 years of age		
	**Total population** (n=3994)	**TG2A negative** (TG2A <7 U/ml) (n=3940)	**TG2A positive** (TG2A >7 U/ml) (n=54)	p-value[a]
**Child characteristics[b]**				
Birth weight (grams; mean, SD)	3434 (551.7)	3433.1 (551.0)	3493.7 (604.6)	0.42
Female gender (n,%)	1934 (48.4)	1899 (48.2)	35 (64.8)	**0.01**
Ethnicity (n,%)				
Non-Western	1312 (33.7)	1300 (33.9)	12 (22.6)	0.09
Western	2578 (66.3)	2537 (66.1)	41 (77.4)	
Breastfeeding (n,%)				
Exclusively breastfed in the first 4 months	656 (25.8)	645 (25.7)	11 (30.6)	0.15
Partially breastfed in the first 4 months	1650 (64.9)	1625 (64.8)	25 (69.4)	
Never breastfed	237 (9.3)	237 (9.5)	0 (0.0)	
Gluten introduction >6 months (n,%)	1498 (56.1)	1479 (56.2)	19 (51.4)	0.55
Diet score (standardized to 1200 kcal/d; mean, SD)	4.2 (1.3)	4.2 (1.3)	4.0 (1.3)	0.48
Vitamin D supplementation (n,%)	1131 (45.0)	1113 (44.9)	18 (50.0)	0.54
Gastroenteritis past year (n, %)	1375 (40.0)	1352 (39.9)	23 (47.9)	0.26
BMI child at 6 years of age (kg/m^2; median, IQR)	15.8 (15.1-16.9)	15.9 (15.1-16.9)	15.4 (14.7-16.3)	**0.01**
Playing outside at 6 years of age (hours/day; median, IQR)	1.3 (0.8-2.1)	1.3 (0.8-2.1)	1.3 (0.7-2.1)	0.70
25(OH)D serum concentrations at birth (nmol/L; median, IQR)	28.9 (15.2-46.6)	28.9 (15.2-46.6)	33.6 (13.7-45.9)	0.84
Season of blood draw at 6 years of age (n,%)				
Winter/Spring	1989 (49.8)	1956 (49.6)	33 (61.1)	0.09
Summer/Fall	2005 (50.2)	1984 (49.7)	21 (38.9)	
25(OH)D serum concentrations at 6 years of age (nmol/L; median, IQR)	63.9 (45.1-81.0)	63.9 (45.1-81.0)	62.0 (44.9-82.7)	0.80
25(OH)D status at 6 years of age (n,%)				
Deficient (<50 nmol/l)	1199 (30.0)	1182 (30.0)	17 (31.5)	0.35
Sufficient (≥50 to <75 nmol/L)	1463 (36.6)	1448 (36.8)	15 (27.8)	
Optimal (≥75 nmol/L)	1332 (33.4)	1310 (33.2)	22 (40.7)	
Risk allele frequencies vitamin D deficiency variants				
CYP2R1 - GG (n,%)	1080 (40.9)	1066 (41.0)	14 (35.9)	0.66
DHCR7 - GG (n,%)	460 (11.5)	455 (17.5)	5 (12.8)	0.12
GC - TT (n,%)	1459 (36.5)	1441 (55.4)	18 (46.2)	0.51
CYP24A1 - TT (n,%)	1538 (38.5)	1516 (58.2)	22 (56.4)	0.59
HLA DQ2 and/ or DQ8 presence (n,%)	1105 (41.8)	1070 (41.1)	35 (89.7)	**<0.001**

**Maternal characteristics[b]**				
Maternal educational level (n,%)				
Low(≤secondary education)	1893 (52.2)	1874 (52.3)	19 (41.3)	0.14
High (≥higher education)	1736 (47.8)	1709 (47.7)	27 (58.7)	
Maternal smoking during pregnancy (n,%)	551 (15.9)	545 (15.9)	6 (15.0)	0.87
Maternal 25(OH)D serum concentrations second trimester pregnancy (nmol/L; median, IQR)	50.3 (27.3-76.0)	50.3 (27.3-76.1)	51.8 (25.0-75.5)	0.82

Values are frequencies (percentages), means (standard deviations (SD)) or medians (interquartile range (IQR)) from the observed dataset

[a] p-values are calculated with the Chi-square test for categorical variables, Mann-Whitney U test for non-normally distributed variables and Student's T test for continuous normal distributed variables and reflect differences between TG2A positive and negative groups. Fisher's exact test was obtained in case the expected count was less than five. A p-value <0.05 was considered significant.

[b] Data was missing for birth weight (0.2%), ethnicity (2.6%), breastfeeding (36.3%), gluten introduction (33.2%), diet score (53.7%), vitamin D supplementation (37.0%), gastroenteritis past year (13.9%), BMI child at 6-year visit (0.2%), playing outside at 6 y (28.3%), 25(OH)D serum concentrations at birth (45.5%), genes associated with vitamin D deficiency (33.9%), HLA DQ2/DQ8 presence (33.8%), maternal smoking during pregnancy (13.4%), maternal 25(OH)D serum concentrations second trimester pregnancy (23.9%)

Supplementary Table 2. Associations between TG2A positivity (binary independent variable) and serum 25(OH)D concentrations (continuous dependent variable) at 6 years of age (crude data)

	25 (OH) D concentrations (nmol/L)		
**TG2A concentrations U/ml**	**Crude model** β (95% CI)	**Model 1** aβ (95% CI)	**Model 2** aβ (95% CI)
**TG2A negative** n=3940	*Reference*	*Reference*	*Reference*
**TG2A positive** n=54	-2.20 (-9.73;5.33)	-2.50 (-9.05;4.06)	-3.09 (-12.20;6.02)

Values (β's) are based on linear regression models and reflect between group differences in serum 25 (OH) D concentrations (nmol/L) at 6 years of age relative to the reference group.
*Denotes statistical significance (P <0.05)
Model 1 was adjusted for season of blood draw (winter/spring and summer/autumn) and ethnicity (Western and non-Western). Model 2 was adjusted for season of blood draw, ethnicity, 25 (OH) D concentrations mother during pregnancy (nmol/L), birth weight (grams), gender, and breastfeeding exclusiveness (yes/no).
Abbreviations: β, beta; aβ, adjusted beta; CI, confidence interval.

Supplementary Table 3. Associations between TG2A positivity (binary independent variable) and serum 25(OH)D concentrations (continuous dependent variable) at 6 years of age (imputed data)

	25 (OH) D concentrations (nmol/L)							
**TG2A concentrations U/ml**	**Crude model** β (95% CI)	**Model 1** aβ (95% CI)	**Model 2** aβ (95% CI)	**Model 3** aβ (95% CI)	**Model 4** aβ (95% CI)	**Model 5** aβ (95% CI)	**Model 6** aβ (95% CI)	**Model 7** aβ (95% CI)
**TG2A negative** *n=3940*	*Reference*	*Reference*	*Reference*	*Reference*	*Reference*	*Reference*	*Reference*	*Reference*
**TG2A positive** *n=54*	-2.20 (-9.72;5.33)	0.08 (-6.97;7.12)	-2.20 (-8.73;4.33)	0.06 (-6.92;7.04)	-0.10 (-3.69;3.49)	-0.13 (-7.12;6.92)	0.26 (-6.78;7.30)	-1.73 (-8.31;4.85)
	**25 (OH) D concentrations (nmol/L)**							
**TG2A concentrations U/ml among HLA DQ2 and/or HLA DQ8 positive children**	**Crude model** β (95% CI)	**Model 1** aβ (95% CI)	**Model 2** aβ (95% CI)	**Model 3** aβ (95% CI)	**Model 4** aβ (95% CI)	**Model 5** aβ (95% CI)	**Model 6** aβ (95% CI)	**Model 7** aβ (95% CI)
**TG2A negative** *n=1070*	*Reference*	*Reference*	*Reference*	*Reference*	*Reference*	*Reference*	*Reference*	*Reference*
**TG2A positive** *n=35*	-1.17 (-10.43;8.09)	0.31 (-8.29;8.91)	-2.11 (-10.14;5.91)	0.30 (-7.76;8.36)	0.87 (-7.72;9.46)	0.35 (-8.29;8.98)	0.45 (-8.15;9.05)	-1.19 (-9.05;6.66)

Values (β's) are based on linear regression models and reflect between group differences in serum 25 (OH) D concentrations (nmol/L) at 6 years of age relative to the reference group.
Multivariable model 1 was adjusted for season of blood draw (winter/spring versus summer/autumn). Model 2 was adjusted for season of blood draw (winter/spring versus summer/autumn) and ethnicity (Western versus non-Western). Model 3 was adjusted for season of blood draw (winter/spring versus summer/autumn) and 25 (OH) D concentrations of the mother during pregnancy (nmol/L). Model 4 was adjusted for season of blood draw (winter/spring versus summer/autumn) and birth weight (grams). Model 5 was adjusted for season of blood draw (winter/spring versus summer/autumn) and gender. Model 6 was adjusted for season of blood draw (winter/spring versus summer/autumn) and breastfeeding exclusiveness (yes/no). Model 7 was adjusted for all covariates.
Abbreviations: β, beta; aβ, adjusted beta; CI, confidence interval.

Supplementary Table 4. Associations between TG2A positivity (binary independent variable) and serum 25(OH)D concentrations (continuous dependent variable) at 6 years of age (crude data)

	**25 (OH) D concentrations (nmol/L)**							
**TG2A concentrations U/ml**	**Crude model** β (95% CI)	**Model 1** aβ (95% CI)	**Model 2** aβ (95% CI)	**Model 3** aβ (95% CI)	**Model 4** aβ (95% CI)	**Model 5** aβ (95% CI)	**Model 6** aβ (95% CI)	**Model 7** aβ (95% CI)
**TG2A negative** n=3940	*Reference*	*Reference*	*Reference*	*Reference*	*Reference*	*Reference*	*Reference*	*Reference*
**TG2A positive** n=54	-2.20 (-9.72;5.33)	0.08 (-6.97;7.12)	-2.50 (-9.05;4.06)	0.52 (-7.24;8.28)	-0.10 (-7.13;6.94)	-0.13 (-7.18;6.92)	-1.12 (-9.61;7.37)	-3.09 (-12.20;6.02)

Values (β's) are based on linear regression models and reflect between group differences in serum 25 (OH) D concentrations (nmol/L) at 6 years of age relative to the reference group.
Multivariable model 1 was adjusted for season of blood draw (winter/spring versus summer/autumn). Model 2 was adjusted for season of blood draw (winter/spring versus summer/autumn) and ethnicity (Western versus non-Western). Model 3 was adjusted for season of blood draw (winter/spring versus summer/autumn) and 25 (OH) D concentrations mother during pregnancy (nmol/L). Model 4 was adjusted for season of blood draw (winter/spring versus summer/autumn) and birth weight (grams). Model 5 was adjusted for season of blood draw (winter/spring versus summer/autumn) and gender. Model 6 was adjusted for season of blood draw (winter/spring versus summer/autumn) and breastfeeding exclusiveness (yes/no). Model 7 was adjusted for all covariates.
Abbreviations: β, beta; aβ, adjusted beta; CI, confidence interval.

Supplementary Table 5a. Associations between TG2A positivity (binary independent variable) and serum 25(OH)D concentrations (continuous dependent variable) at 6 years of age, selected by mean diet quality score <4.17 (imputed data)

	25 (OH) D concentrations (nmol/L)		
**TG2A concentrations U/ml**	**Crude model** β (95% CI)	**Model 1** aβ (95% CI)	**Model 2** aβ (95% CI)
**TG2A negative** *n=925*	*Reference*	*Reference*	*Reference*
**TG2A positive** *n=14*	-7.84 (-23.07;7.38)	-5.14 (-19.41;9.14)	-3.75 (-17.13;9.63)

Values (β's) are based on linear regression models and reflect between group differences in serum 25 (OH) D concentrations (nmol/L) at 6 years of age relative to the reference group.
Multivariable model 1 was adjusted for season of blood draw and ethnicity (Western versus non-Western). The main model was adjusted for season of blood draw (winter/spring versus summer/autumn), 25 (OH) D concentrations mother during pregnancy (nmol/L), birth weight (grams), gender, ethnicity (Western versus non-Western) and breastfeeding exclusiveness (yes/no).
Abbreviations: β, beta; aβ, adjusted beta; CI, confidence interval.

Supplementary Table 5b. Associations between TG2A positivity (binary independent variable) and serum 25(OH)D concentrations (continuous dependent variable) at age 6 years, selected by mean diet quality score ≥4.17 (imputed data)

	**25 (OH) D concentrations (nmol/L)**		
**TG2A concentrations U/ml**	**Crude model** β (95% CI)	**Model 1** aβ (95% CI)	**Model 2** aβ (95% CI)
**TG2A negative** *n=899*	*Reference*	*Reference*	*Reference*
**TG2A positive** *n=10*	9.09 (-7.70;25.88)	8.42 (-6.86;23.70)	8.06 (-7.20;23.32)

Values (β's) are based on linear regression models and reflect between group differences in serum 25 (OH) D concentrations (nmol/L) at 6 years of age relative to the reference group.
Multivariable model 1 was adjusted for season of blood draw and ethnicity (Western versus non-Western). The main model was adjusted for season of blood draw (winter/spring versus summer/autumn), 25 (OH) D concentrations mother during pregnancy (nmol/L), birth weight (grams), gender, ethnicity (Western versus non-Western) and breastfeeding exclusiveness (yes/no).
Abbreviations: β, beta; aβ, adjusted beta; CI, confidence interval.

Supplementary Table 6. Associations between TG2A positivity (binary independent variable) and serum 25(OH)D concentrations (continuous dependent variable) at age 6 years, tested for effect modification by risk allele variants for vitamin D deficiency (n=3994)(crude data)

	**25 (OH) D concentrations (nmol/L)**			
**TG2A concentrations U/ml**	**Model 1** p-value for interaction	**Model 2** p-value for interaction	**Model 3** p-value for interaction	**Model 4** p-value for interaction
**TG2A negative** *n=3940*	0.33	0.68	0.62	0.25

*A p-value <0.05 was considered significant.
Model 1 shows possible interaction between rs2282679 and TG2A concentrations. Model 2 shows possible interaction between rs6013897 and TG2A concentrations. Model 3 shows possible interaction between rs10741657 and TG2A concentrations. Model 4 shows possible interaction between rs12785878 and TG2A concentrations.
